# Optical properties of cicada wings covered by graphene studied by nano‐Raman spectroscopy

**DOI:** 10.1111/jmi.70044

**Published:** 2025-11-12

**Authors:** Vitor A. F. Torres, André Pereira, Diego Sier, Rafael Nadas, Jane Elisa Guimarães, Rayan Alves, Renato Veloso, Fernanda Brant, Bernardo R. A. Neves, Ado Jorio

**Affiliations:** ^1^ Departamento de Física Universidade Federal de Minas Gerais Belo Horizonte Minas Gerais Brazil; ^2^ FabNS Parque Tecnológico de Belo Horizonte‐BHTec Belo Horizonte Brazil; ^3^ Institut für Physik Humboldt‐Universität zu Berlin, Newtonstraße 15 Berlin Germany

**Keywords:** AFM, cicada wings, graphene, nanometric surfaces, TERS

## Abstract

Some biological systems exhibit nanoscale constructions to produce optical effects. This study utilises Atomic Force Microscopy (AFM) and Tip‐Enhanced Raman Spectroscopy (TERS) to study the complex bionanometric structure of cicada wings. Topographical irregularities of the wings due to α‐chitin nanopillars hinder the probe's approach to the sample, a crucial step in overcoming the light diffraction limit in TERS measurements. To mitigate this issue, graphene was deposited, promoting surface smoothing and ensuring a reliable TERS measurement. Combined analyses of AFM and TERS mapping revealed a significant enhancement of the graphene 2D band, particularly in the regions surrounding the nanometric pillars, while the characteristic α‐chitin Raman peaks are evident on top of the pillars, clarifying details of how light passed through the material.

## 1 INTRODUCTION

Biological systems have long employed nanoscale constructions to produce optical effects.^1^, ^2^, ^3^ The optical transmission of cicada wing structures is notably high, reaching approximately 90% across a broad wavelength range from 450 to 2500 nm.^4^ The cicada *Carineta fasciculata*,^5^ a species widely found in South America, exhibits ordered arrangements on its wings, similar to those observed in other species. These roughly periodic arrangements consist of conical papillary structures with a roughly hexagonal pattern in domains of approximately 1 $\mu m^{2}$–2 $\mu m^{2}$, present on both the dorsal (upper) and ventral (lower) surfaces of the cicada wing.^6^ These structures are responsible for the anti‐reflective function associated with camouflage.^7^, ^8^ The mechanism behind the transparency of cicada wings is explained as *impedance matching*. When light propagates from one medium to another, part of it is reflected at the interface between the two media. The amount of reflected light depends on the disparity between the optical impedances of the two media. If the optical impedances are equal, that is, if there is impedance matching, all the light is transmitted, and no reflection occurs. Furthermore, the conical shape of these protrusions contributes to the hydrophobicity of the wings and their self‐cleaning effect.^9^, ^10^ These results have inspired studies in which the wings were used as a substrate for surface‐enhanced Raman spectroscopy (SERS), with the wings prepared with thin silver films to enhance the local electric field on the surface.^11^, ^12^, ^13^ Considering this context, Raman spectroscopy emerges as a potential technique for analysing the nanostructures and properties of cicada wings. However, the diffraction limit of light, as presented by Ernst Abbe, imposes a restriction on resolution, where the minimum discernible distance between two light emitters is on the order of half the wavelength of the incident light.^14^, ^15^

**FIGURE 1:**
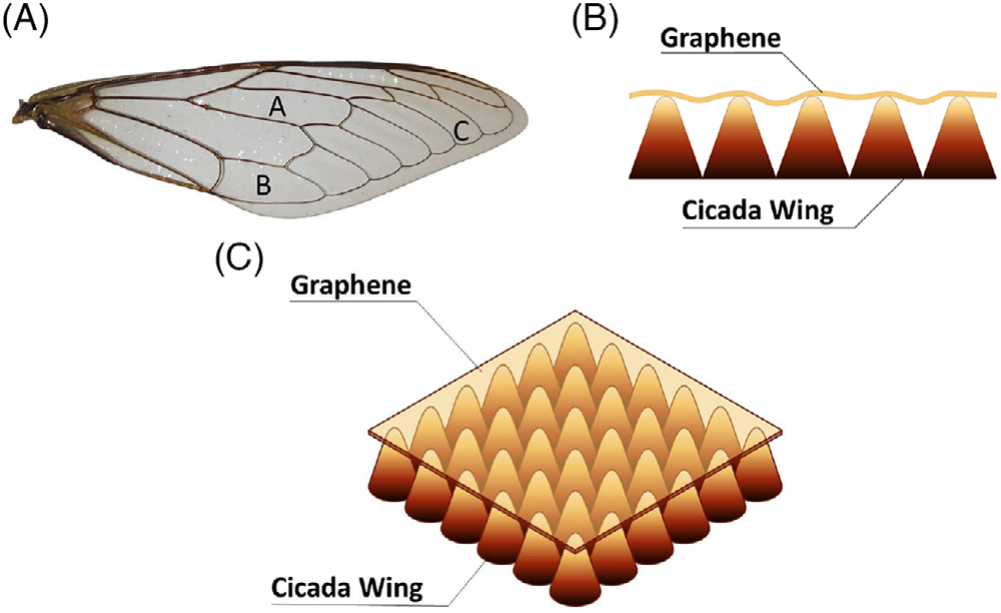
(A) Photo of the cicada wing used in the graphene deposition process. Regions with different nanometric heights are indicated by specific (A, B or C) markers. (B) Representation of the periodic structures of cicada wings with deposited graphene. (C) Illustrative three‐dimensional scheme of graphene deposition on the pillars present on the surface of the cicada wing.

In this study, we analysed the conical papillary structures of cicada wings using tip‐enhanced Raman spectroscopy (TERS), a technique that combines the chemical sensitivity of Raman spectroscopy with the nanoscale resolution of scanning probe microscopy (SPM).^16^, ^17^ To achieve this objective, a two‐dimensional material, graphene, was deposited on specific regions of the surface, as illustrated in Figure 1. This deposition enables the measurements to be performed, as graphene resulted in a significant smoothing of the topography, in addition to acting as a guide for the optimisation of the TERS signal.^18^ Additionally, TERS‐integrated intensity maps revealed that the graphene G and 2D bands exhibit higher intensity in the regions between the pillars, whereas the characteristic wing peaks are distributed both over the pillars and in the adjacent regions.

## 2 EXPERIMENTAL DETAILS

### 2.1 Sample preparation

Insect cuticle performs crucial functions such as mechanical protection, morphological definition, and waterproofing, serving as an exoskeleton.^19^ Cicada wings are an extension of the cuticular exoskeleton, featuring a network of veins that provides rigid structural support, with α‐chitin, protein, and lipids as their main chemical components.^20^ In this study, cicada wings from the species *Carineta fasciculata* were extracted from deceased insects and subjected to a cleaning process. Initially, the wings were placed in a beaker containing acetone and treated using an ultrasonic cleaning procedure with a LimpSonic device for 5 min. The acetone was then replaced with ethanol, and the ultrasonic process was repeated. To ensure the removal of any remaining impurities, the wings were washed with deionised (DI) water for the same duration in the ultrasonic system.^12^

Once the wings were properly cleaned, graphene preparation for deposition was initiated. The chemical vapour deposition (CVD) technique enables the large‐scale fabrication of graphene through the decomposition of hydrocarbons at high temperatures, leading to the deposition of carbon on the surface of a catalyst.^21^ Copper was used as the catalyst since it has a low chemical affinity for carbon, allowing the carbon nucleation process on the copper surface to be controlled, forming only a single atomic layer of carbon, that is, graphene.^22^ To transfer the graphene onto the cicada wing, the graphene and copper substrate was coated with poly(methyl methacrylate) (PMMA). This polymer facilitates the mechanical handling of graphene during the transfer process, preventing mechanical damage due to stress. The copper removal was carried out through chemical etching, where the copper, graphene and PMMA sample was left floating in an ammonium persulphate solution for 12 h.^23^

After this period, all the copper was completely removed, leaving only the PMMA and graphene structure. Using a flat support, the 16 ${\text{mm}}^{2}$ sample was carefully extracted from the ammonium persulphate solution and subjected to successive deionised (DI) water baths to clean the underside of the graphene. The sample was then left to rest for 12 h. Subsequently, the graphene and PMMA sample was transferred onto the cicada wing surface. The PMMA removal was performed in two stages: first using acetone, followed by isopropyl alcohol, and finally dried with a nitrogen jet.^24^ The result of the deposition process is illustrated schematically in Figure 1B and C.

### 2.2 Nano‐Raman and topography

The equipment used for the micro‐ and nano‐Raman measurements was the Porto laboratory prototype, equipped with a Shamrock 303i spectrometer operating with a 600 l/mm grating and absolute spectral resolution of 2 ${\text{cm}}^{-1}$.^25^ This system performs spectroscopy with nanometric resolution and spectral mapping, generating 2D images with 24 nm pixels for the studied sample, enabling the identification of chemical and structural variations on the surface.^26^ To conduct the measurements, the system employs a HeNe laser with a radially polarised excitation, wavelength λ=632.8nm. The beam is guided through a series of optical elements before illuminating the sample, using an inverted microscope equipped with a 50x air objective, featuring a numerical aperture (NA) of 0.60 and a working distance (WD) of 11 mm. The measurement system integrates a confocal Raman microscope with an Atomic Force Microscopy (AFM) scanning head positioned at the top.

Porto AFM uses a tuning fork operating in non‐contact mode, characterised by the absence of direct contact between the tip and the sample, with a resonance frequency close to 32 kHz. The TERS probe is fabricated through a two‐step lithography process that produces gold structures consisting of a micropyramidal trunk with a nanopyramidal tip. The size of the nanopyramid can be tuned to achieve resonance with the laser wavelength. This geometry is referred to as a Plasmon‐Tunable Tip Pyramid (PTTP),^27^ whose spatial resolution is determined by the diameter of the nanoantenna apex, typically below 30 nm. A gold PTTP, tuned to the HeNe laser (632.8 nm), is attached to one of the tuning fork tines and positioned approximately 5 nm from the sample.^16^ After interacting with the laser, the nanoantenna generates a locally enhanced near‐field TERS signal.^28^, ^29^ The topographic images obtained by AFM and the maps were plotted using Gwyddion software (v2.66), while the processing of Raman spectra and TERS intensity maps was performed using PortoFlow software by FabNS (v1.21).

The antireflective property of the cicada wing plays a fundamental role in enabling measurements using the Porto laboratory prototype, as it ensures that the laser can penetrate the sample while its field is amplified by the TERS tip. This antireflection behaviour can be analysed on surfaces with thicknesses in the nanometre to micrometre range using thin film theory.^9^, ^30^, ^31^ For the special case of normal incidence, the Fresnel equation used to calculate the reflection and transmission of incident light takes the form

(1) $$R={\left(\frac{n_{\text{wing}}-n_{\text{air}}}{n_{\text{wing}}+n_{\text{air}}}\right)}^{2},$$

and calculations indicate a reflection close to 5%, considering the refractive index of air ($n_{\text{air}}$ = 1) and the refractive index of the wing ($n_{\text{wing}}$), which is essentially equivalent to the refractive index of α‐chitin ($n_{\text{wing}}$ = $n_{\text{chitin}}$ = 1.61).^32^ Although the deposition of graphene causes a slight modification in this index, such an alteration is disregarded due to the extremely small thickness of graphene, approximately 0.335 nm.^33^ To minimise reflection, the thickness *d* of the thin film must be adjusted so that the waves reflected at the two interfaces cancel out by destructive interference, satisfying the condition

(2) $$\begin{array}{c} 2n_{\text{wing}}d=\frac{\lambda }{2}\;⟶\;d=\frac{\lambda }{4n_{wing}}. \end{array}$$

Thus, the ideal height of the cicada wing's nanometric arrangements is given by Equation (2), where λ is the wavelength of the HeNe laser used. The calculated optimal thickness to minimise reflection is approximately 100 nm.

**FIGURE 2:**
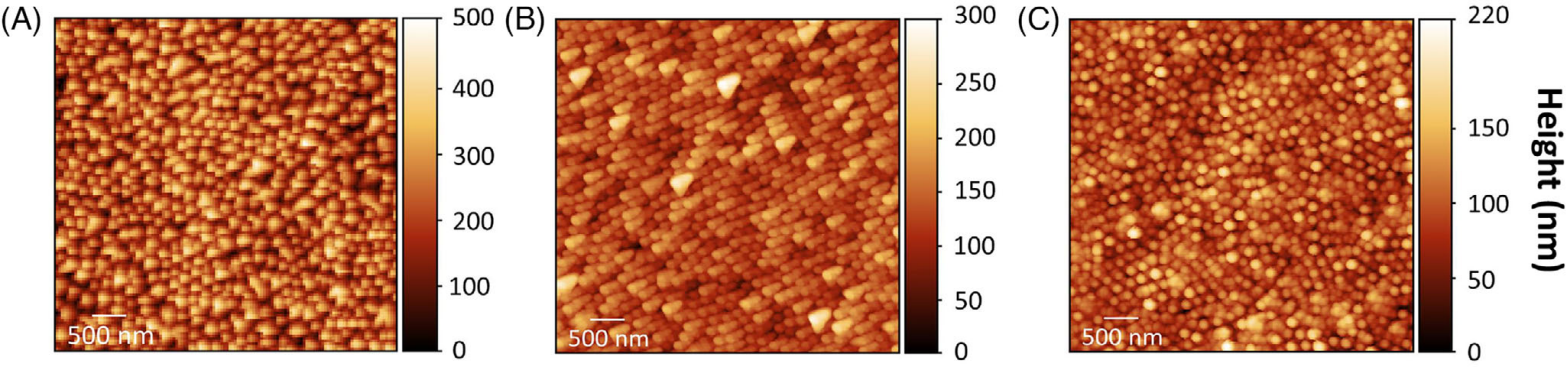
AFM images of cicada wings, with scan areas of 5 × 5 $\mu m^{2}$. (A) Region A, located at the centre of the wing. (B) Region B, situated in the lower part of the wing. (C) Region C, corresponding to the tip of the cicada wing. Here A, B and C are locations according to Figure 1A.

Surface topography characterisations were also performed using a Park XE‐70 system in three distinct regions of the cicada wings, as shown in Figure 2, corresponding to regions A (wing centre), B (lower part), and C (wing tip), located in Figure 1A.

The AFM analysis is presented in Table 1. In each region, two measurements were performed, totalling an area of 50 $\mu m^{2}$ for determining the average heights. The average spacings between the pillars were obtained from centre‐to‐centre measurements of 60 pillars in each region. The values presented in Table 1 correspond to the average heights and average spacings between the pillars, along with their respective uncertainties. For these analyses, HQ:NSC/No Al cantilevers, suitable for delicate measurements, were employed, operating in intermittent contact mode with a tip–sample set point of 12 nm and a tip approach–retract force (Z servo gain) of 1.100 (arb. units).

**TABLE 1:** Different regions of the cicada wing, as indicated in Figure 2, with their average heights and average pillar spacing. The uncertainties associated with both measurements were obtained from the standard deviation.

Wing region	Average heights (nm)	Average inter‐pillar spacing (nm)
A	218±9	199±4
B	123±6	182±3
C	88±6	201±4

**FIGURE 3:**
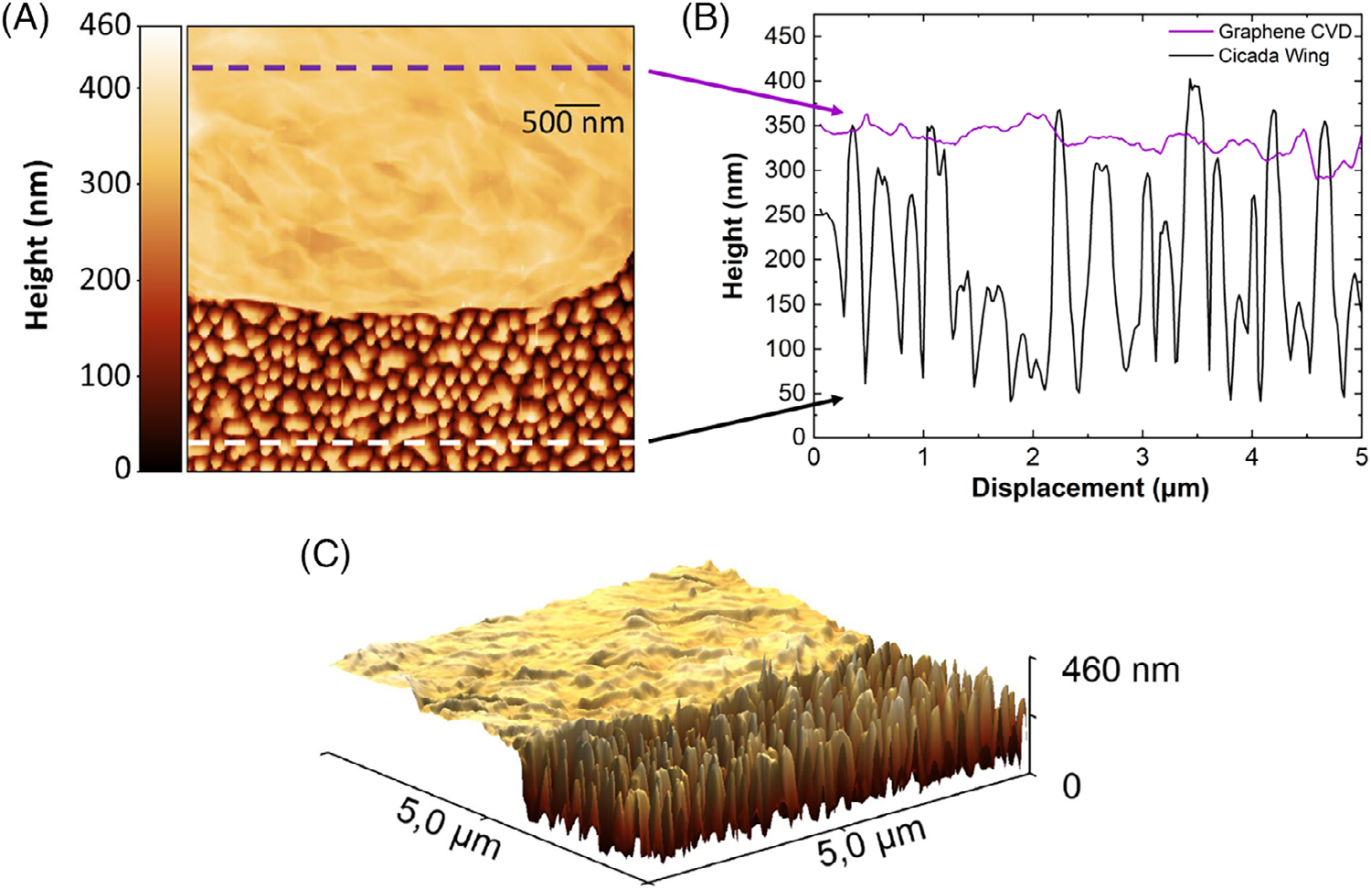
AFM measurements of cicada wing partially covered by graphene, on a scan area of 5 × 5 $\mu m^{2}$, consisting of 256 pixels per line. (A) AFM topography at the interface between CVD graphene and the cicada wing surface. The dashed lines in the upper and lower sections indicate the regions from which line profiles were extracted. (B) Topographic line profile along an axis, comparing the height of the CVD graphene surface (purple line) and the cicada wing (black line). (C) Three‐dimensional (3D) representation of the AFM data, highlighting the interface between CVD graphene, characterised by a homogeneous surface, and the cicada wing, composed of papillary conical structures.

Graphene deposition was carried out on segments of regions A and B. These regions, despite hosting the tallest structures, are the topographically flattest areas of the sample. Graphene was chosen as a two‐dimensional material for two main reasons: (i) its ease of production and (ii) the well‐known Raman signal.^34^ The deposition was successful, resulting in a smoothing of the wing surface and allowing the clear distinction of the interface between the wing and graphene, as illustrated in Figure 3. The root mean square (RMS) roughness, determined using the Gwyddion software from the mean height deviations relative to a reference line, showed a significant reduction, decreasing from approximately 25 nm in the regions without graphene to about 7 nm in the regions covered with graphene (see Figure 3 for reference). Additionally, the topographic profile revealed a significant reduction in the height of the nanostructures after deposition. While the original surface of the cicada wing exhibits abrupt variations, the region covered with graphene exhibited a significantly more uniform topography, as evidenced in Figure 3B. This result is crucial as it enables the TERS tip to approach the sample homogeneously at a distance of approximately 5 nm, allowing direct interaction with the surface and overcoming the diffraction limit of light.

Graphene also acts as a guide; by being deposited over the nanostructures of the wing, its Raman signal can be maximised through alignment and focusing, enabling the acquisition of the TERS signal from the cicada wing structure.^18^

## 3 RESULTS AND DISCUSSION

### 3.1 Graphene and cicada wing spectral characteristics

**FIGURE 4:**
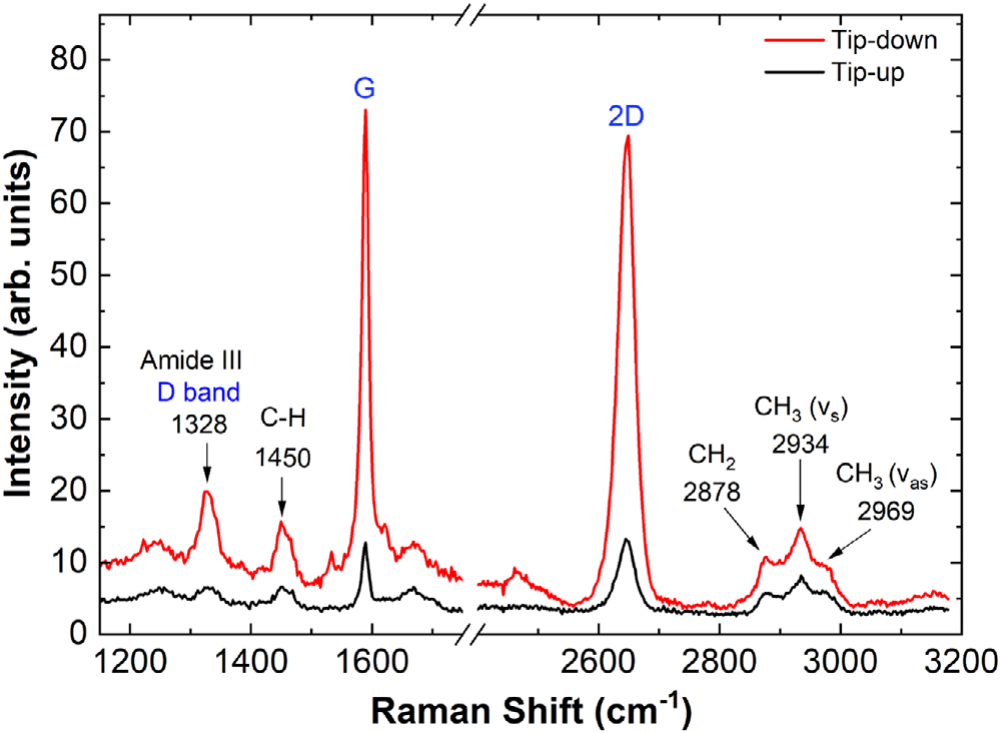
Tip‐up (black) and tip‐down (red) spectra of the cicada wing with deposited graphene, acquired using a HeNe laser at a power of 0.76mW at the sample, with five accumulations of 5 s each. The most intense α‐chitin peaks, highlighted in black, and the D, G, and 2D bands of graphene, in blue, are emphasised. The omitted region corresponds to a silent spectral range.

Figure 4 shows the typical micro‐Raman (tip‐up) and nano‐Raman (tip‐down, TERS) spectra of the graphene‐cicada wing system. The relevant graphene bands for this study correspond to well‐known peaks in the literature: the G‐band peak (∼1580 ${\text{cm}}^{-1}$), which originates from a first‐order Raman scattering process associated with the stretching of the carbon‐carbon (C‐C) bond; the 2D‐band peak (∼2700 ${\text{cm}}^{-1}$), which is a characteristic second‐order Raman scattering signature of ${\mathrm{sp}}^{2}$ carbon materials.^34^ Since the 2D peak exhibits higher intensity, our analysis here will mostly focus on this Raman feature.

The analysed cicada wing structure is composed of α‐chitin, which features an antiparallel arrangement of its polymer chains, providing structural stability to the wing. The Raman spectrum of α‐chitin represents the relevant bands associated with the cicada wing, with the following prominent peaks identified (see Figure 4): the peak corresponding to the Amide III band, around 1328 ${\text{cm}}^{-1}$, which represents the stretching vibration of the carbon‐nitrogen (C‐N) bond, along with the angular deformation of the nitrogen‐hydrogen (N‐H) bond, and provides information about the structural organisation of Amide III in α‐chitin; the peak around 1450 ${\text{cm}}^{-1}$, resulting from the angular deformation of carbon‐hydrogen (C‐H) bonds, where hydrogen atoms move relative to the carbon plane to which they are attached; the peaks corresponding to the symmetric stretching vibrational modes of the aliphatic methylene $\left(-{\text{CH}}_{2}-\right)$ groups at approximately 2878 ${\text{cm}}^{-1}$ and methyl $\left(-{\text{CH}}_{3}\right)$ groups around 2934 ${\text{cm}}^{-1}$; the asymmetric stretching vibrational mode of the methyl $\left(-{\text{CH}}_{3}\right)$ group near 2969 ${\text{cm}}^{-1}$.^35^ These aliphatic groups do not absorb visible light, which enhances the transparency of the cicada wing.^36^

### 3.2 Tip‐enhancement characteristics for graphene and cicada wing

To clarify the differences between micro‐Raman (tip‐up) and nano‐Raman (tip‐down, TERS) spectroscopy of the graphene‐cicada wing system, we performed tip‐down and tip‐up measurements (red and black spectra in Figure 4, respectively). In the tip‐down configuration, there is a combined contribution from both the near‐field and far‐field, whereas in the tip‐up configuration only the far‐field contribution is obtained. Data processing using Principal Component Analysis (PCA) allowed for pattern identification and a significant reduction in signal noise.^37^ Since graphene is synthesised via CVD, the presence of the D band, associated with defects in the material's structure, is usually expected.^38^ This peak, located at approximately 1350 ${\text{cm}}^{-1}$, overlaps with the signal from the Amide III band, as observed in Figure 4.

**FIGURE 5:**
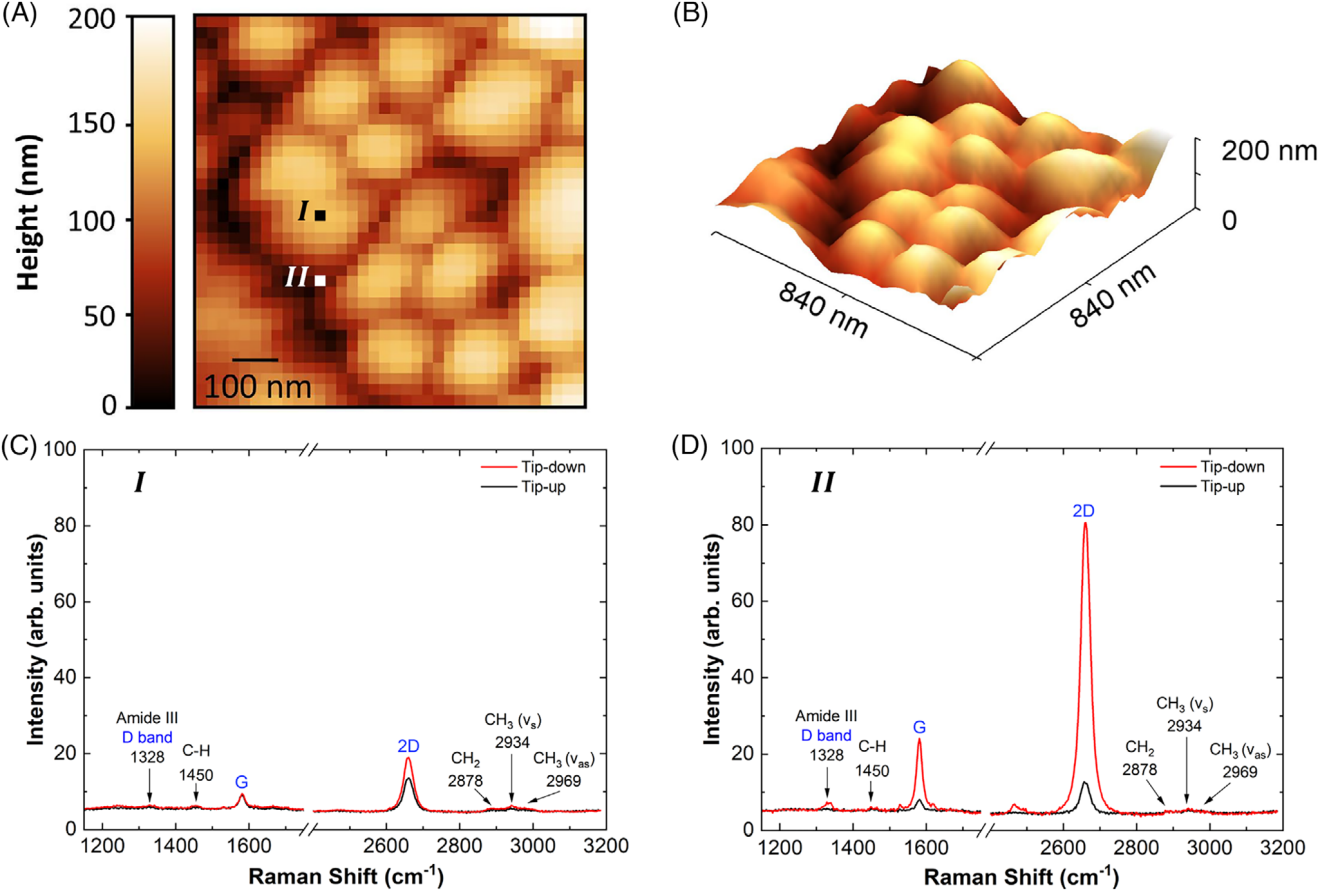
AFM and TERS measurements of the graphene‐cicada wing system. The scan window was 840 × 840 ${\mathrm{nm}}^{2}$, with 36 pixels per line and an accumulation time of 1 s per pixel. (A) AFM image with regions I and II highlighted, indicating the locations where Raman spectra in (C, D) were acquired. (B) Three‐dimensional diagram representing the distribution of pillars on the cicada wing covered with graphene, corresponding to the AFM data in (A). (C, D) Tip‐down (red) and tip‐up (black) spectra from regions I and II, respectively, highlighting the graphene bands labeled in blue and the characteristic α‐chitin peaks labeled in black.

Moreover, the topographic response (AFM) was obtained during the TERS measurement, as shown in Figure 5A and B. Additionally, the spatial relationships between the pillars and their nearest neighbours were maintained when in comparison to the uncoated counterpart (see Figure 3A), with an average distance of approximately 200 nm between pillars. This result enables a detailed analysis of the spectral response on top of the cicada wing pillars and in the open space between pillars, as evidenced in the point spectra presented in Figure 5C and D, obtained respectively from points I and II indicated in Figure 5A. Region I represents the top of the wing structures, white region II represents the valley in between the wing structures. Figure 5D presents a Raman spectrum obtained from a region between pillars, where a significantly more intense near‐field Raman signal from graphene is observed.

The spectral enhancement factor $F_{\text{TERS}}=A_{\text{tip-down}}/A_{\text{tip-up}}$ is used to quantify the increase in the Raman signal resulting from the interaction between the tip and the sample, where $A_{\text{tip-down}}$ represents the integrated intensity (area) of a Raman peak in the tip‐down configuration, and $A_{\text{tip-up}}$ corresponds to the equivalent value in the same region under the tip‐up configuration. The analysis of $F_{\text{TERS}}$ to both the most intense graphene band (2D) and the most intense cicada wing peak (methyl group symmetric ${\mathrm{CH}}_{3}$, at 2934 ${\mathrm{cm}}^{-1}$) shows an interesting result: For the graphene 2D peak, the enhancement is considerably higher outside the pillars. For the cicada Raman feature, the opposite behaviour is observed, that is, the enhancement is higher on top of the pillars (see Table 2). It is important to notice that $F_{\text{TERS}}$ values greater than 2.0 indicate the presence of a near‐field effect, while for $F_{\text{TERS}}\leq 2$.0 it is not clear whether the enhancement is simply associated with a mirror effect, where the apex of the TERS probe reflects back to the detector the forward scattering. The reasoning behind these observations is discussed in the next section.

**TABLE 2:** Spectral enhancement factor $F_{\text{TERS}}=A_{\text{tip-down}}/A_{\text{tip-up}}$ on the pillar region and outside the pillars for the two prominent Raman features from graphene and cicada wing. The mean values and their respective uncertainties were determined from the analysis of six‐point spectra obtained around the regions I and II highlighted in Figure 5A.

Raman peaks	On the pillar	Outside the pillar
2D band	1.2±0.1	7.6±0.2
Methyl group symmetric $CH_{3}$	2.6±0.1	1.8±0.1

### 3.3 Nano‐Raman spectral mapping of the graphene‐cicada wing system

**FIGURE 6:**
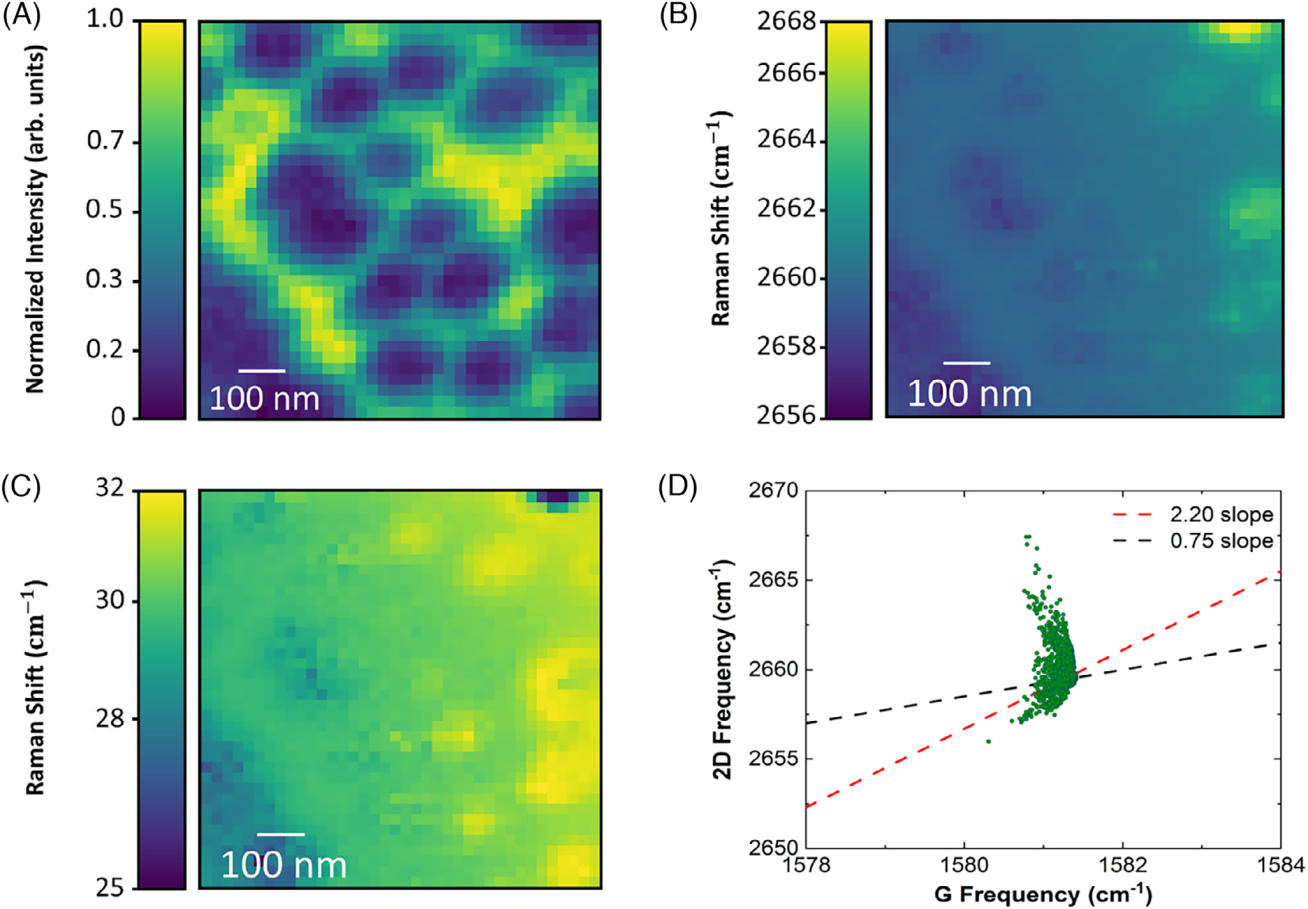
TERS maps of graphene 2D band. (A) Normalised mapping obtained by TERS for the integrated intensity (area). (B) Frequency map. (C) Full width at half maximum (FWHM) map. (D) $\omega_{\text{2D}}$ versus $\omega_{G}$ plot, composed of points extracted from the area map.

TERS maps of the graphene 2D band are shown in Figure 6. This measurement is colocalised with the AFM shown in Figure 5A, with a pixel size of 24 nm. Graphene is deposited across the entire analysed area, coating the nanostructures. Consistent with the result in Table 2, the TERS map in Figure 6A exhibits higher integrated intensities in the areas between the pillars. This result can be rationalised considering that the higher TERS intensities of graphene are concentrated in the regions of greater light transmission, which happens in the spacing between the pillars. Quantitative analysis showed that the integrated intensity of transmitted light, after normalisation to the respective area, was approximately four times greater through the interpillar than through the pillar area.

Still about graphene, in addition to the analysis based on Raman intensities, it is known that the relationship between the frequency of the 2D band ($\omega_{\text{2D}}$) and the frequency of the G band ($\omega_{G}$) provides information about localised strain and doping.^39^, ^40^, ^41^ The $\omega_{\text{2D}}$ varies linearly with $\omega_{G}$ under mechanical strain and doping, with slopes that are distinct: 2.20 for hydrostatic strain and 0.75 for doping.^41^ The distribution of $\omega_{\text{2D}}$ versus $\omega_{G}$ values observed in our experiments (green dots in Figure 6D) reveals a significantly more pronounced variation in the frequency of the 2D band. In the right‐hand region of the AFM image in Figure 5A, the pillars exhibit larger heights, as evidenced by the colour scale, and show a direct correlation with the recorded frequency values in Figure 6B, where the highest frequencies were observed. Figure 6C shows that the tallest pillar exhibited the lowest full width at half maximum (FWHM), in contrast to the other tall pillars, which displayed higher FWHM values. These results suggest that the dispersion of the 2D band is intrinsically related to the presence of deformation in the structure. Although this behaviour bears some resemblance to distributions characteristic of uniaxial strain^42^ or to the strong interaction of graphene with metals,^43^, ^44^ our results show only a 2 ${\text{cm}}^{-1}$ variation in the G band, which is distinct from the behaviour reported in the cited works.

**FIGURE 7:**
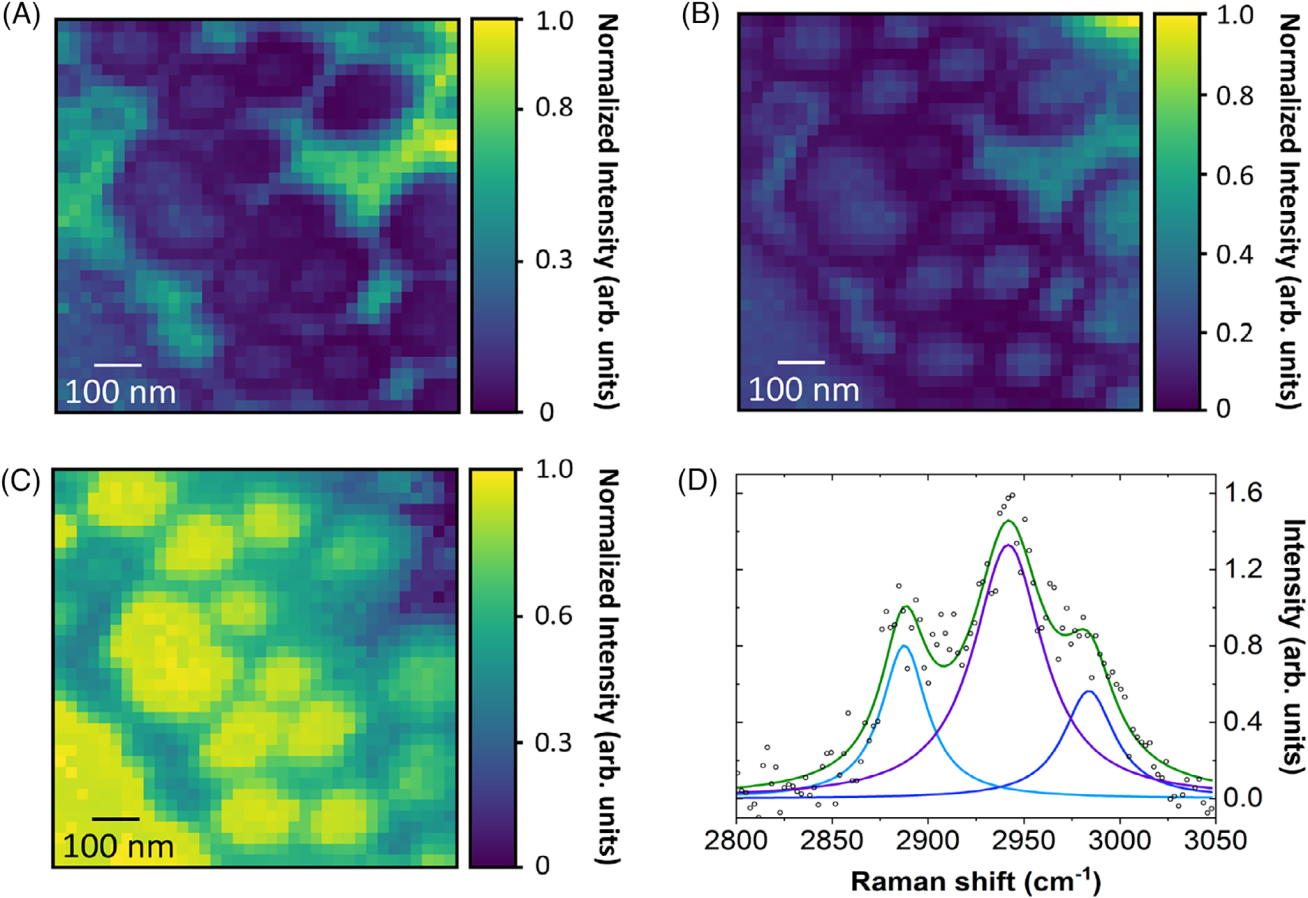
TERS intensity maps of cicada wing. (A) The vibrational class associated with Amide III overlapped with the peak of the graphene D band (∼1328 ${\text{cm}}^{-1}$); (B) the angular deformation of the (C–H) bond (∼1450 ${\text{cm}}^{-1}$); (C) the methyl group in symmetric stretching mode (∼2934 ${\text{cm}}^{-1}$), as defined in (d). (D) Spectral representation of the 2800–3050 ${\mathrm{cm}}^{-1}$ feature fitted with Lorentzians, used to define the peaks associated with the aliphatic functional group of the wing. For (A) and (B), Lorentzian curves were also applied in the spectral fitting, not shown due to the simplicity of the line profiles.

Going further on the spectral analysis, Figure 7A highlights the normalised intensity spatial distribution of peaks corresponding to Amide III overlapped with the graphene D band (∼1328 ${\text{cm}}^{-1}$). Figure 7B, in turn, stand for the angular deformation of the C–H bond (∼1450 ${\text{cm}}^{-1}$), exhibiting null TERS signal intensity at the projection of the base of the nanostructures. In this figure, the normalised signal intensity is concentrated both in the region of the pillars and in the areas corresponding to the trenches. Finally, Figure 7C displays the intensity of the methyl group in symmetric stretching mode (∼2934 ${\text{cm}}^{-1}$, see peaks in Figure 7D), which is intense on top of the pillar structures, consistent with the values of Table 2.

These results show that, while the graphene peaks are more intense in the regions between pillars, where light propagates freely, as shown in Figure 6, the peaks related to the cicada wing are more intense in the pillar structures, which are the locations where the TERS tip can reach the cicada wing structure. This rationale suggests that the TERS features related to the Amide III, which is known to be convoluted with the D band of graphene, as shown in Figure 7A, are likely dominated by the graphene D peak. The methylene functional group $\left(-{\text{CH}}_{2}-\right)$ exhibits a less clear hybrid profile, probably because this feature is actually too weak, hidden in the noise, only detectable after the PCA analysis. In contrast, the peaks associated with the methyl $\left(-{\text{CH}}_{3}\right)$ functional group, shown in Figure 7C, exhibit a well‐defined behaviour, with higher intensities when the TERS tip is directly over the conical structures of the wing.

## 4 CONCLUSION

The ability to generate spectral maps is a fundamental aspect of Raman spectroscopy application. Tip‐enhanced Raman spectroscopy (TERS) becomes especially relevant when significant differentiation between nanometric‐sized locations cannot be observed in a micro‐Raman analysis. The results discussed here indicate a direct relationship between the presence of nanometric pillars and the integrated intensity of the characteristic α‐chitin peaks in the cicada wing.

The deposition of CVD‐grown graphene resulted in a significant reduction in the topographical roughness of the natural cicada wing nanostructures, while preserving the spatial relationships between adjacent pillars. The topographic profile graph revealed that before deposition, there was a variation of approximately 360 nm between the highest structure and the deepest valley; after graphene deposition, this variation was reduced to about 80 nm in the analysed region. This transformation converted an initially irregular morphology, unsuitable for TERS measurements, into a more homogeneous topography compatible with the scanning capabilities of the TERS tip.

The near‐field enhancement provided by the tip‐down configuration enabled a clear distinction between graphene bands and the characteristic peaks of the cicada wing, as observed in the TERS integrated intensity maps. Interestingly, using TERS on cicada wings, we demonstrated that light travels majorly through the spaces between the pillars.

The present study did not focus on revealing new biological aspects; instead, it provided robust evidence of the feasibility of employing non‐trivial natural surfaces in chemical and surface characterisation techniques, demonstrating potential for application to other biological systems. Further challenges remain for long‐term and dynamic measurements. The future implementation of ultrastable^45^ and high‐speed^46^ systems offers a promising pathway to applying this technique in the study of biomimetic materials and complex nanodevices, where real‐time chemical and structural characterisation is essential.

## CONFLICT OF INTEREST STATEMENT

The authors declare no conflict of interest.
